# The role of adipose and muscle tissue breakdown on interorgan energy substrate fluxes in a *Pseudomonas aeruginosa* induced sepsis model in female pigs

**DOI:** 10.14814/phy2.70129

**Published:** 2025-01-03

**Authors:** Ryan Morse, Gabriella A. M. Ten Have, John J. Thaden, Marielle P. K. J. Engelen, Sarah Rice, Martin Hagve, Nicolaas E. P. Deutz

**Affiliations:** ^1^ Center for Translational Research in Aging and Longevity, Department of Health and Kinesiology Texas A&M University College Station Texas USA; ^2^ Department of Gastrointestinal Surgery University Hospital North‐Norway Tromso Norway

**Keywords:** amino acid metabolism, gluconeogenesis, glucose, lipid metabolism, pig, sepsis

## Abstract

Sepsis leads to an acute breakdown of muscle to support increased caloric and amino acid requirements. Little is known about the role of adipose and muscle tissue breakdown and intestinal metabolism in glucose substrate supply during the acute phase of sepsis. In a translational porcine model of sepsis, we explored the across organ net fluxes of gluconeogenic substrates. In 13 pigs, acute sepsis was induced by IV infusion of *Pseudomonas aeruginosa*, while in 9 pigs saline (control) was given for 18 h. Blood samples were collected between 12 and 18 h and analyzed with HPLC and LCMS. In sepsis, glucose plasma concentration was reduced (*p* = 0.0028). A concordant increase in splanchnic area net release of glucose (*p* = 0.0049), due to reduced uptake in the portal drained viscera (PDV) (*p* = 0.0032) with an unchanged liver production (*p* = 0.7861). The hindquarter showed a higher release of alanine (*p* = 0.0002), glutamine (*p* = 0.003), and lactate (*p* = 0.0007), but not for glycerol (*p* = 0.5718). Diminished PDV uptake of gluconeogenic amino acids, increased liver uptake of these substrates (*p* < 0.05), while no change in liver glycerol uptake (*p* = 0.3170), did not lead to an increased net liver glucose release. In the acute phase of sepsis, we hypothesize an important role of altered intestinal amino acid metabolism and breakdown of muscle proteins, but not of glycolysis to support gluconeogenesis.

## 1 INTRODUCTION

Sepsis is a severe generalized inflammatory condition that results in organ dysfunction (Cecconi et al., 2018). The mortality rate of this condition is around 20% (Fleischmann et al., 2016). Those who are able to recover from the underlying infection and inflammatory response, characteristic of sepsis, are often left with significant long‐term impairments (Cecconi et al., 2018). One of the key pathologies of this condition is a catabolic response in which the lean muscle tissue of the body is broken down (Deutz et al., 2021; Mira et al., 2017).

In critically ill humans, muscle protein breakdown was found to be increased, while muscle protein synthesis only gradually increased over time (Liebau et al., 2021). In animals, the liver shows a significant increase in the uptake of amino acids, consistent with the increased acute phase protein production (Ten Have, Hommen et al., 2017) (Strnad et al., 2017). However, limited knowledge of the changes in interorgan amino acid fluxes in relation to energy substrate fluxes is available.

In healthy physiological conditions, adipose tissue, as an essential source of physiological energy storage, helps to provide sufficient glucose via the gluconeogenesis pathway with glycerol (Melkonian et al., 2021) and seems to protect breaking down substantial quantities of muscular tissue for glucogenic substrates like the amino acids glutamine and alanine (Cahill Jr., 1970). Sepsis is characterized by a substantial increase in protein breakdown which increases the whole‐body production of the glucogenic substrates (Deutz et al., 2021; Hasselgren & Fischer, 1999; Leverve, 1998). While several studies indicate that the inflammation in sepsis triggers lipolysis and therefore the release of the glucogenic substrate glycerol to the system (Muniz‐Santos et al., 2023), it is unclear if this occurs in conjunction with the severe protein breakdown in the first acute phase in sepsis.

Glycerol is utilized in the liver as one of the substrates contributing to gluconeogenesis along with lactate and glucogenic amino acids like alanine and glutamine. Previous research showed that these substrates make up over 90% of the overall glucogenic precursor (Gerich, 1993). The aim of our study was to examine glucose production by the liver and the kinetics of glucogenic substrates in the acute phase of sepsis. Pigs have been well established as a good model of physiological derangements in sepsis, particularly in study of the hypermetabolic state (Bruins et al., 2003) (Goldfarb et al., 2005). Therefore in the present study, we measured in a translational *Pseudomonas aeruginosa*‐induced model of sepsis in catheterized pigs (Ten Have et al., 2018) (Ten Have et al., 1996) the energy substrate fluxes across organs, including the liver. We measured arterial concentrations and organ net fluxes of glycerol, alanine, glutamine, glucose, and lactate. The results of the present study provided insights into the relationships between available energy substrate fluxes in the acute septic state. This knowledge is critical for the development of new and improved nutritional strategies in the acute phase of sepsis.

## 2 MATERIALS AND METHODS

### 2.1 Animals

Twenty‐seven female Yorkshire crossbred pigs aged 8–12 weeks and weighing 20–25 kg were obtained from a commercial breeder. At least 1 week before surgical catheter implantation, the animals were allowed to adapt to individual housing in 2 × 3 m galvanized bar runs equipped with drinking nipples and isolated floors. The room was maintained at 22–25°C and 55% humidity. A normal day‐night cycle was maintained by lighting the enclosures for 12 h per day (07:00–19:00). Pigs were administered a food intake of 1 kg/day of Harlan Tecklad vegetarian pig/sow grower (Harlan Laboratories, Indianapolis, IN, USA). This study was approved by the Institutional Animal Care and Use Committee (IACUC) of University of Arkansas Medical Sciences (Little Rock, AR, USA).

### 2.2 Surgical catheter implantation

We implanted catheters surgically as described in previous publications (Schooneman et al., 2015; Ten Have et al., 1996). Briefly, a midline laparotomy was performed, through which catheters were placed for blood sampling in the abdominal aorta, the portal, hepatic, caval, and renal veins. We also implanted a catheter in the abdominal aorta and splenic vein for the infusion of para‐aminohippuric acid (PAH) to allow for the measurement of plasma flow. An additional central venous catheter was implanted to allow for the infusion of medication and fluids both before and during the sepsis experiment.

We provided standard preoperative and postoperative care as described previously (Ten Have et al., 2018) (Ten Have et al., 1996). Briefly, the investigator and an assigned veterinarian closely monitored the anesthesia and surgical recovery. Analgesia was maintained postsurgically with 1 mg/kg body weight of flunixin meglumine (Flunixamine®, Zoetis, CA). We administered 187.5 mg of Lincomycin hydrochloride monohydrate and 250 mg of Spectinomycin sulfate tetrahydrate (Linco‐Spectin®, Zoetis, CA) diluted in 20 mL of saline twice daily for 4 days via the central venous catheter postsurgery for infection prophylaxis. During the recovery period, animals were accustomed to a small moveable cage (0.9 × 0.5 × 0.3 m) that was subsequently used to perform experiments on conscious animals.

### 2.3 Experimental design

The experiment began after 7–10 days of postsurgical recovery. Of the twenty‐seven animals, five were lost due to postsurgical complications. The experiments were started at 15:00 h on the experimental day, 6 h after the last food intake (half of the daily amount: 0.5 kg). Animals were selected for the sepsis or control group in a balanced randomized fashion. At time point *t* = −1 h, a continuous infusion of PAH (para‐aminohippuric acid, Sigma‐Aldrich, USA), was started (25 mM) at 60 mL/h into the splenic vein, and 60 mL/h into the inferior vena cava for measurement of plasma flow as described previously (Schooneman et al., 2015). At *t* = 0 h, sepsis was induced in 13 pigs by the start of a continuous intravenous infusion of *Pseudomonas aeruginosa* (PA) at a rate of 10^9^ CFU/mL/hr. (Ten Have et al., 2018) (freshly cultured from a batch culture original from IRS 12‐4‐4, Shriners burn Institute, University of Texas Medical Branch, Galveston; original from a burn patient at Brook Army Medical Center in San Antonio, TX, tested for virulence, antibiotic resistance, viability). This dose was selected to ensure that animal hemodynamics were kept in the expected ranges for sepsis and did not result in septic shock. A solution of 0.9% NaCl in the same volume was given to 9 pigs (control). Fluid resuscitation with 0.9% NaCl was started at the same time as the PA infusion at a rate of 30 mL/kg body weight/hour. No food was administered during the experiment. General appearances, as well as hemodynamic measurements, were monitored and recorded continuously. Blood samples were taken from arterial, venous, portal, and hepatic catheters at times *t* = −2, −0.5, 0, 11.5, 14, 16, 17, 17.5, and 18 h.

### 2.4 Blood sample processing

Blood samples were collected in lithium‐heparinized tubes and directly placed on ice. These samples were centrifuged within 1 h at 8000*g* for 5 min at 4°C. For the measurement of glucose and glycerol concentrations, the heparinized plasma was collected and frozen at −80°C using liquid nitrogen for later analysis. For the analysis of amino acid concentrations, 500 μL of plasma was transferred to a tube containing 50 μL of a solution of 33% w/w trichloroacetic acid solution, thoroughly mixed, and stored at −80°C for later analysis.

### 2.5 Laboratory analysis

#### 2.5.1 PAH analysis

For measurement of the plasma flow, PAH concentrations in TCA‐deproteinized plasma samples were compared with PAH standards using microplate‐based assay of PAH using *p*‐dimethylaminocinnamaldehyde (Sigma‐Aldrich, USA), which produces a red color on reaction with PAH (Agarwal, 2002; Schooneman et al., 2015).

#### 2.5.2 Amino acid analysis

We determined amino acid (AA) concentrations on a fully automated LC–MS/MS system (QTRAP 5500 MS with Ekspert microLC 200 LC, SCIEX, Foster City, CA). For AA concentration measurements we added 20 μL of the supernatant of TCA‐deproteinized plasma to 20 μL of internal standard, a mixture of a high mass stable isotope of every AA. Within 3 days before the LC–MS/MS analysis, we derivatized the samples together with external standards at concentrations within the physiological range (calibration curve of concentration) with 9‐fluorenylmethoxycarbonyl (Fmoc) chloride. After neutralization, we injected 160 nL of the solution onto a 0.5 mm × 100 mm micro LC column packed with 2.7 μm, 90‐A HALO C18 beads (SCIEX, Foster City, CA) kept at 50°C. We eluted analytes with a segmentally linear gradient from 35% to 85% acetonitrile in water supplemented with ammonium acetate to 15 μM and 5% isopropanol. We used electrospray triple quadrupole tandem mass spectrometry in multiple reaction monitoring (MRM) mode for detection. We fragmented the Fmoc AA derivatives in the collision cell for detection of either free aminoacyl anions or a fragment larger by 26 atom mass units (coming from the Fmoc derivative) to have the highest sensitivity. We simultaneously used mass analyses for all AA and its internal standards. We integrated the mass signal areas to enable AA concentration calculations using a calibration curve.

#### 2.5.3 Glucose and glycerol analysis

Glucose and glycerol were simultaneously measured using a GC–MS/MS system (Bruker, a CP‐8400 autosampler, a 436‐GC gas chromatograph with a programmed temperature vaporizing (PTV) inlet, and a Scion TQ triple quadrupole mass spectrometer with a chemical ionization (CI) source, all controlled by MS Work Station v. 8.2.1 software).

Acetylation: In preparation for the GC–MS, samples were acetylated as follows: To 20 μL of plasma were added 20 μL of an internal standard aqueous solution of glycerol(1,1,2,3,3‐D_5_) and glucose(U‐^13^C_6_) and 250 μL of ice‐cold acetone, after which samples were vortexed and placed at −20°C for at least 15 min. Samples were centrifuged at 45,000*g* and 4°C for 5 min. The supernatant was poured into a 10 × 75 mm disposable borosilicate culture tube (14‐958‐10A, ThermoFisher, Waltham, MA). 250 μL of water and 250 μL of n‐hexane were added, after which the sample tubes were capped (#60828–706, VWR, Radnor PA) and gently rocked end‐to‐end on a horizontal rocker platform for 15 min. Phases were separated by centrifuging for 5 min at 2000*g*. The upper organic phase, as well as any interphase, was discarded, with glycerol and glucose remaining in the aqueous phase. Samples were dried down using a Speed‐Vac (SC210A, ThermoFisher, Waltham, MA) set on high heat for 90 min, then no heat until samples were completely dry. For acetylation, 15 μL of fresh 1:2 pyridine–acetic anhydride was added and tubes were then vortexed briefly to dissolve dried residues and then centrifuged at 2000*g* for 30 s to collect the liquid at the bottom of the tube. Samples were reacted at 60°C for 10 min on a heat block. The end result of this reaction is to derivatize glycerol into triacetin and glucose into glucose pentaacetate to allow for quantification by gas chromatography. Samples were then lightly dried for 20 min in the Speed‐Vac with no rotor heating. To the reacted and dried samples were added 1 mL of ethyl acetate after which the samples were vortexed and transferred to threaded 2 mL borosilicate autosampler vials (11–1056, Sciencix, Burnsville, MN). Vials were closed with septum caps (11–1091, Sciencix, Burnsville, MN) and positioned on the autosampler tray.

Gas chromatography: Of each derivatized sample, 0.6 μL was injected into a 2 mm i.d. bottom‐constriction liner (#23463, Restek) maintained at 200°C by the PTV inlet with its split vent closed, and thus onto an initially cold (53°C) 30 m × 0.25 mm (0.15 μm film) ZB‐50 column (Phenomenex, Torrance, CA). Sample thus recondensed at the column front was freed of ethyl acetate solvent by 40 psi pressure pulse of the helium carrier gas. After 0.35 min, the oven was rapidly heated to 125°C and at 0.45 min the helium was reset to a 2 mL/min constant flow rate for analysis. The analytical gradient was bilinear, initially ramping at a rate of 15°C/min to 163°C, eluting glycerol at 3.5 min, then after rapid heating to 250°, ramping again at 15°C/min to 273°, eluting glucose at 5.5 min. The column was reconditioned at 320° for 0.25 min then cooled at a maximal rate to 53°.

Mass spectrometry: After a 3 min solvent delay to conserve the source filament, molecules eluting from the GC were negatively ionized by chemical ionization using methane at 15 psi as reagent gas. Ions were subjected to collision‐activated dissociation (CAD) using argon as the CAD gas at a pressure of 2.0 mTorr, and collision energies of 12.0 and 16.5 eV, respectively, for glycerol and glucose. Selected reaction monitoring (SRM) was used first to measure glycerol and glycerol(D_5_) by their m/z 159 > 57 and 164 > 61 collisional breakages, respectively, then of glucose (211 > 109) and glucose(^13^C_6_) (217 > 115). Fragments were detected through an extended dynamic range detector. Peaks for glycerol, glucose, and their internal standard isotopes were integrated using MS Work Station 8.21. Areas were converted to area ratios within Google Sheets. For external standard results, area ratios were plotted in GraphPad Prism against the standards' known concentrations to yield glycerol and glucose calibration curves, which were then used to convert sample area ratios to plasma concentrations.

#### 2.5.4 Lactate analysis

Lactate concentrations in the heparinized porcine plasma samples were measured using a well‐established 2nd generation enzymatic, colorimetric assay (Cobas c111, Roche Diagnostics, Mannheim, Germany).

### 2.6 Calculations and statistics

#### 2.6.1 Plasma flow

All calculations were performed using plasma flows and plasma concentrations. The measurement of plasma flows using PAH is described previously (Ten Have et al., 1996).

#### 2.6.2 Transorgan net fluxes

Transorgan net fluxes were calculated using the difference in concentration of the analyzed substances in the arteries leading into the organs of interest and the veins leading out of these organs and plasma flow (Bruins et al., 2000; Ten Have et al., 1996; Wolfe & Chinkes, 2004), using Equation (1).

(1) $$\text{Transorgan}\;\mathrm{net}\;\text{flux}=\mathrm{NF}={\text{Flow}}_{\text{plasma}}\times \left(\left[\text{Out}\right]-\left[\text{In}\right]\right)$$

Equations (2), (3), and (4) are derivations of Equation (1) used for the final calculations of transorgan net fluxes of the portal drained viscera (PDV), splanchnic area (SPL), liver and Hindquarter (HQ). Equation (5) describes the calculation of liver trans organ net balance by subtracting portal‐drained viscera net fluxes from splanchnic net flux. Art, artery; Hep, hepatic vein; Port, portal vein; and Ven, caval vein.

(2) $$\mathrm{NF}\;\mathrm{PDV}={\text{PlasmaFlow}}_{\text{portal}}\times \left(\left[\text{Port}\right]-\left[\mathrm{Art}\right]\right)$$

(3) $$\mathrm{NF}\;\mathrm{SPL}={\text{PlasmaFlow}}_{\text{splanchnic}}\times \left(\left[\mathrm{Hep}\right]-\left[\mathrm{Art}\right]\right)$$

(4) $$\mathrm{NF}\;\mathrm{HQ}={\text{PlasmaFlow}}_{\text{hindquarter}}\times \left(\left[\mathrm{Ven}\right]-\left[\mathrm{Art}\right]\right)$$

(5) $$\mathrm{NF}\;\text{Liver}=\left(\mathrm{NF}\;\mathrm{SPL}\right)-\left(\mathrm{NF}\;\mathrm{PDV}\right)$$

A negative NF value indicates a net uptake, while a positive value indicates a net release.

#### 2.6.3 Statistics

Statistical analyses were performed using Graphpad Prism 10.0. Significance was set at *p* < 0.05. For baseline calculations, average concentrations were used from blood samples collected at time points *t* = −2, −0.5, and 0 h before the start of the PA administration, while the average of samples of time points of *t* = 16, 17, 17.5, and 18 h were used to determine the effects of sepsis. Plasma flow values were described previously (Ten Have et al., 2018).

ANCOVA analysis was used to control for the inherent variation in trans organ flux data (plasma flow, A and V concentrations) to obtain a higher statistical sensitivity. For example, the dependent variable “Portal venous concentration” was adjusted for the following independent variables: individual portal plasma flow, body weight, body temperature, and arterial concentrations of each animal. Predicted Portal venous concentrations were obtained for further calculations. Actual and predicted net fluxes were both calculated (Figure S1).

Arterial concentrations and predicted net organ fluxes were analyzed with a Repeated Measure Two‐way Mixed Effect ANOVA. Post hoc comparisons were done for the following 4 “families” of scientific null‐hypothesis with Uncorrected Fisher's LSD. Null‐ hypothesis 1: There is no difference in baseline measurements between the two randomized experimental groups (Baseline control vs. Baseline sepsis). Null‐hypothesis 2: There is no experimental time effect (Baseline control vs. Post control). Null‐hypothesis 3: There is no sepsis effect (Post control vs. Post sepsis). Null‐hypothesis 4: There is no time effect by sepsis (Baseline sepsis vs. Post sepsis). Significance was set at *p* < 0.05.

Results are presented as mean [95% CI]. When hypothesis 1 was true, one baseline bar was shown in the bar charts. Rejections of the null hypothesis 3 (sepsis‐effect) are presented in the figures. Positive net flux values within the 95% CI indicate a net release of lactate, while negative values indicate a net uptake.

## 3 RESULTS

### 3.1 General remarks

Details regarding the hemodynamic profiles, clinical chemistry, and plasma flow of the animal cohorts can be found in a previous publication (Ten Have et al., 2018). No animals were lost during surgery or sepsis induction, although five animals were lost during the recovery phase of catheter implantation due to intestinal complications. The criteria used for the successful induction of sepsis using PA was based on the diagnostic criteria for SIRS (severe inflammatory response syndrome), which included a body temperature increase of at least two degrees Celsius, heart rate of greater than 90 BPM, and PaCO_2_ of less than 32 mmHg (Kaukonen et al., 2015). Based on these criteria, we have concluded that all pigs that were administered PA were successfully induced into sepsis. Baseline values of the Control and Sepsis experimental groups were comparable.

### 3.2 Arterial concentrations (Figure 1)

**FIGURE 1:**
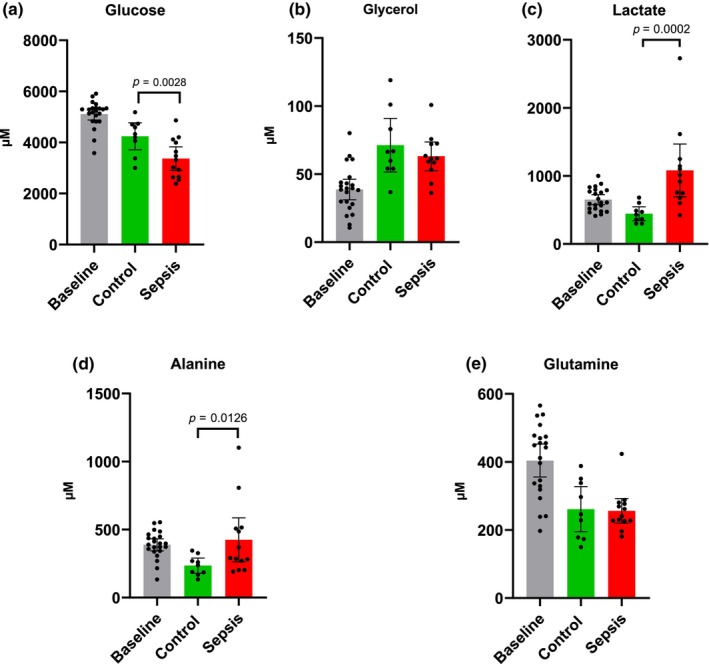
Arterial concentrations. (a) Glucose, (b) glycerol, (c) lactate, (d) alanine, and (e) glutamine. Control *n* = 9 Sepsis *n* = 13. Data are expressed as mean with 95% CI. Statistics. Mixed effect ANOVA. Post hoc uncorrected Fisher's LSD test. Significance (*p* < 0.05) between Control and Sepsis is presented.

Arterial concentrations of glucose showed an overall decrease over time (Two‐Way ANOVA time effect: *p* < 0.0001) and the Sepsis group was lower (*p* = 0.0028) than the Control group (Figure 1a). There was no difference in glycerol concentrations between the control and sepsis groups (Figure 1b), although there was an overall increase (Two‐Way ANOVA Time effect: *p* < 0.0001). Lactate concentrations were higher (*p* = 0.0002) in the sepsis group (Figure 1c) and showed a significant overall time x condition interaction (*p* = 0.0138). An increase in alanine concentrations (*p* = 0.0126) were observed in the sepsis group (Figure 1d). No significant difference was observed between the control and sepsis groups for glutamine (Figure 1e), although both had an overall decrease (Two‐Way ANOVA time effect: *p* < 0.0001).

### 3.3 Glucose net fluxes (Figure 2)

**FIGURE 2:**
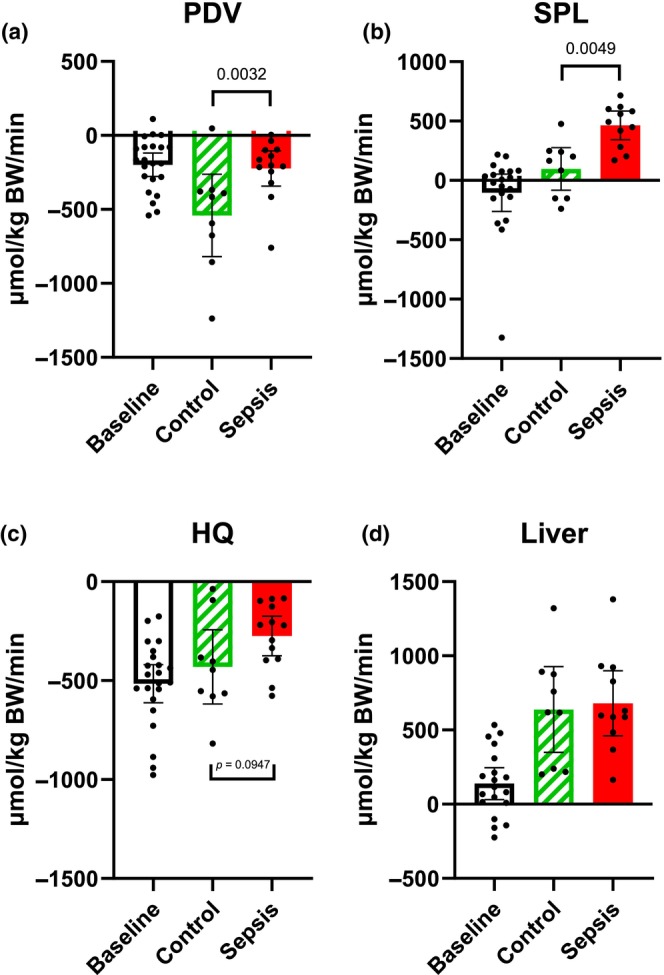
Glucose predicted net flux. (a) The portal drained viscera (PDV), (b) splanchnic area (SPL), (c) hindquarter (HQ), and (d) liver. Positive values within 95% CI indicate a net release of glucose, while negative values indicate a net uptake. Control *n* = 9 Sepsis *n* = 13. Data are expressed as mean with 95% CI. Statistics. Mixed effect ANOVA, Post hoc uncorrected Fisher's LSD test. Significance (*p* < 0.05) between Control and Sepsis is presented.

In the PDV, there was a significant decrease (*p* = 0.0032) in the net uptake of glucose in the sepsis group compared to the control (Figure 2a). In the SPL, there was a significant increase (*p* = 0.0049) in release in the sepsis group compared to the control (Figure 2b). In the HQ, both groups showed a net uptake of glucose, with no overall difference between groups. We only observed a tendency (*p* = 0.0947) of less uptake in the sepsis group (Figure 2c). A net release was observed in the liver, but there was no significant difference between the groups (Figure 2d).

### 3.4 Glycerol net fluxes (Figure 3)

**FIGURE 3:**
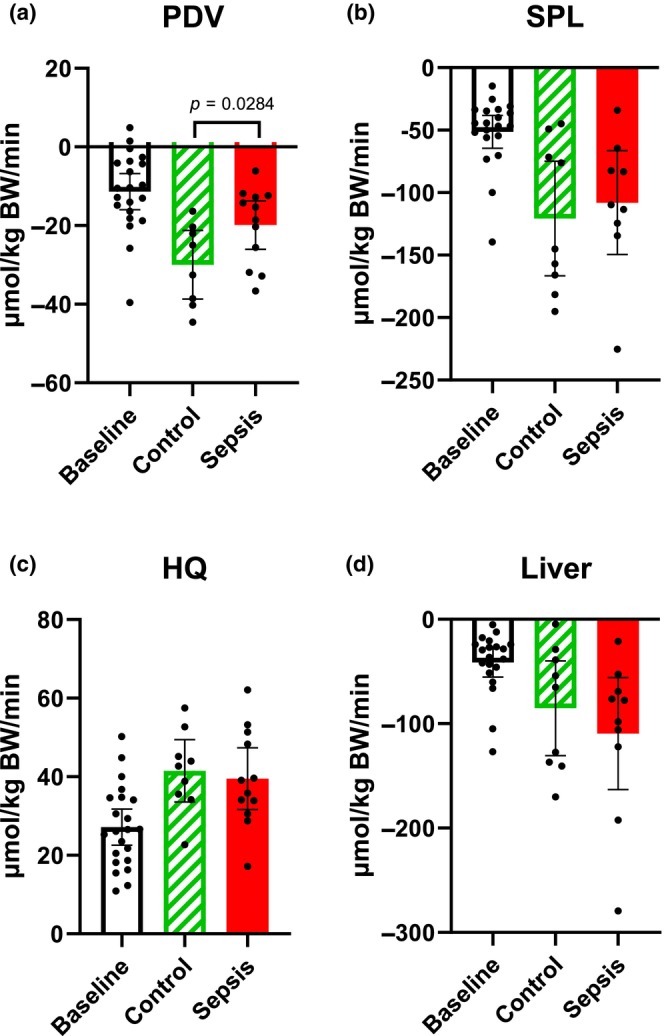
Glycerol predicted net flux. (a) The portal drained viscera (PDV), (b) splanchnic area (SPL), (c) hindquarter (HQ), and (d) liver. Positive values within 95% CI indicate a net release of glycerol, while negative values indicate a net uptake. Control *n* = 9 Sepsis *n* = 13. Data are expressed as mean with 95% CI. Statistics. Mixed effect ANOVA. Post hoc uncorrected Fisher's LSD test. Significance (*p* < 0.05) between Control and Sepsis is presented.

We observed an overall experimental time effect in all across organ measurements (Two‐Way ANOVA time effect: PDV, HQ: *p* < 0.0001, SPL: *p* = 0.0004, Liver: *p* = 0.0022). In the PDV, SPL, and liver, both groups showed a net uptake of glycerol, while in the HQ, a net release. No significant difference in the net flux of glycerol between the control and sepsis groups across the SPL, HQ, or liver, although the uptake in the PDV was less (*p* = 0.0284) in the sepsis group (Figure 3a).

### 3.5 Alanine net flux (Figure 4)

**FIGURE 4:**
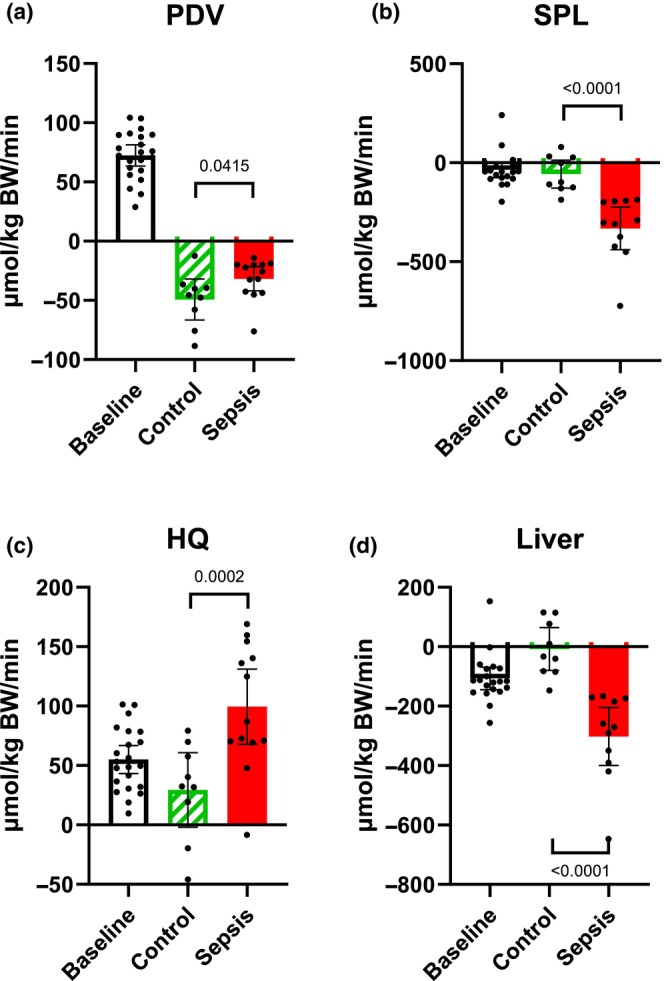
Alanine predicted net flux. (a) The portal drained viscera (PDV), (b) splanchnic area (SPL), (c) hindquarter (HQ), and (d) Liver. Positive values within 95% CI indicate a net release of alanine, while negative values indicate a net uptake. Control *n* = 9 Sepsis *n* = 13. Data are expressed as mean with 95% CI. Statistics: Mixed effect ANOVA. Post hoc uncorrected Fisher's LSD. Significance (*p* < 0.05) between Control and Sepsis is presented.

In the PDV, a reduction (*p* = 0.0415) in the net uptake of alanine was observed in the sepsis group (Figure 4a). In the SPL, the net uptake in the sepsis group was higher (*p* < 0.0001) (Figure 4b). In the HQ, a higher net release of alanine (*p* = 0.0002) was observed in the sepsis group (Figure 4c). In the liver, no net uptake was observed in the control group. This in contrast with the high net uptake (*p* < 0.0001) in the sepsis group (Figure 4d) which was increased compared to baseline uptake (*p* = 0.0015).

### 3.6 Glutamine net flux (Figure 5)

**FIGURE 5:**
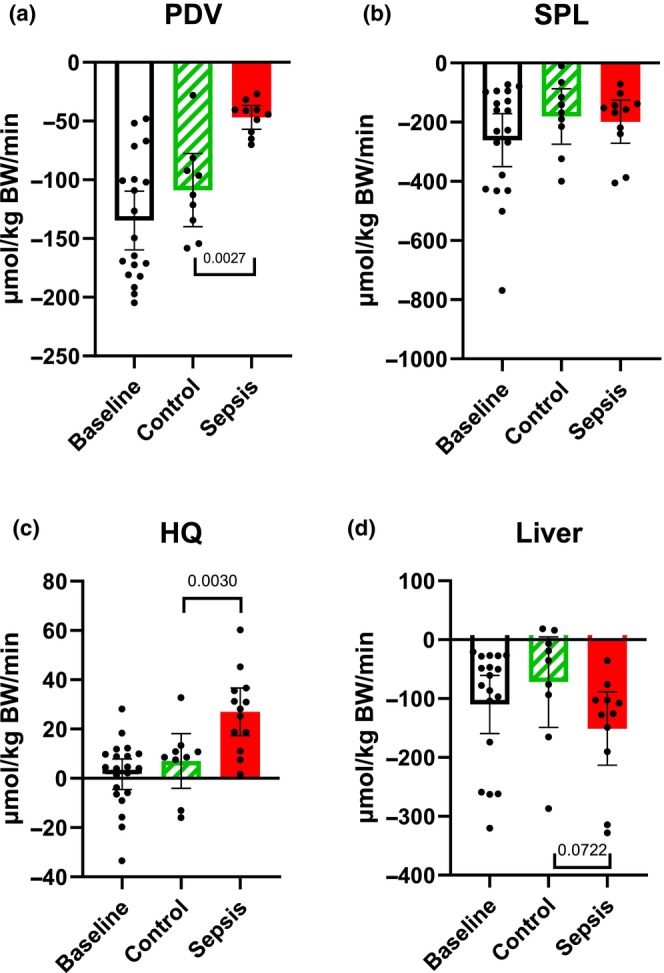
Glutamine predicted net flux. (a) The portal drained viscera (PDV), (b) splanchnic area (SPL), (c) hindquarter (HQ), and (d) liver. Positive values within 95% CI indicate a net release of glutamine, while negative values indicate a net uptake. Control *n* = 9 Sepsis *n* = 13. Data are expressed as mean with 95% CI. Statistics. Mixed effect ANOVA. uncorrected Fisher's LSD. Significance (*p* < 0.05) between Control and Sepsis is presented.

In the PDV, a net decrease (*p* = 0.0027) in the uptake of glutamine was observed between the control and sepsis groups (Figure 5a). Overall, no changes in the net uptake were observed in the SPL. Although a tendency of higher uptake (*p* = 0.0722) in the liver (Figure 5d), in the HQ, a release (*p* = 0.003) was observed in the sepsis group (Figure 5c).

### 3.7 Lactate net flux (Figure 6)

**FIGURE 6:**
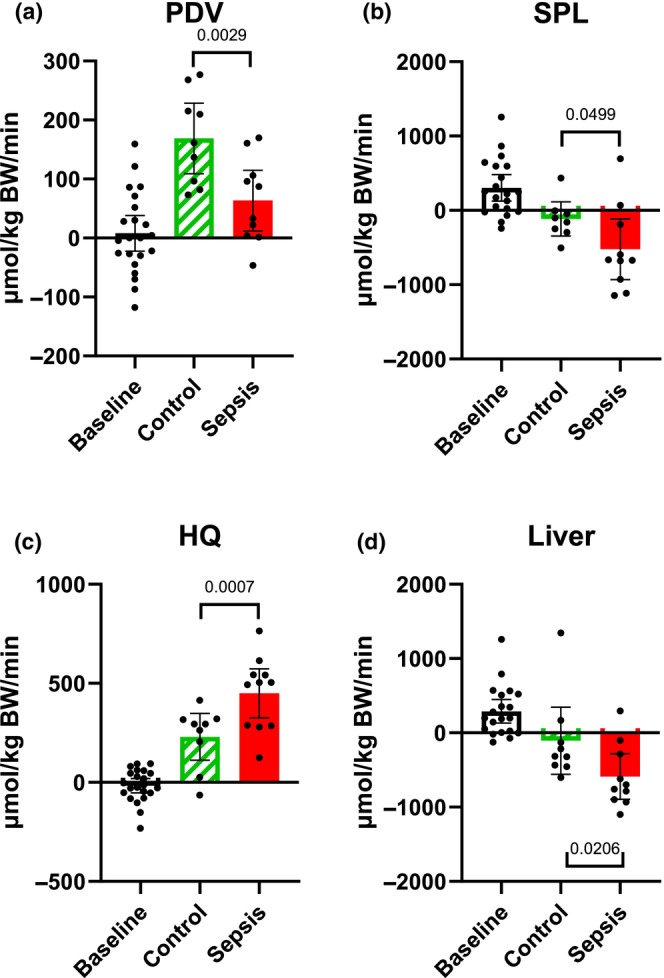
Lactate predicted net flux. (a) The portal drained viscera (PDV), (b) Splanchnic area (SPL), (c) Hindquarter (HQ), and (d) Liver. Positive values within 95% CI indicate a net release of lactate, while negative values indicate a net uptake. Control *n* = 9 Sepsis *n* = 13. Data are expressed as mean with 95% CI. Statistics. Mixed effect ANOVA. post hoc uncorrected Fisher's LSD. Significance (*p* < 0.05) between Control and Sepsis is presented.

In the PDV, the net release of lactate was diminished (*p* = 0.0029) in the sepsis group (Figure 6a). In the SPL, a net uptake (*p* = 0.0499) in the sepsis group (Figure 6b) was observed. In the HQ, a significantly higher net release (*p* = 0.0007) was observed in the sepsis group (Figure 6c). In the liver, a net uptake (*p* = 0.0206) was observed in the sepsis group (Figure 6d).

### 3.8 Cumulative glycogenic substrate fluxes

**FIGURE 7:**
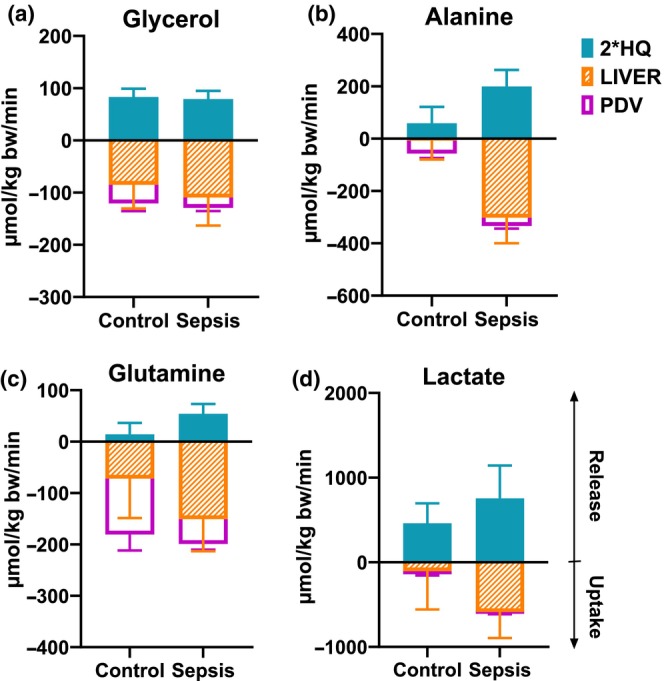
Cumulative organ glycogenic substrate fluxes. (a) Glycerol. (b) alanine, (c) glutamine, (d) lactate. Predicted net fluxes. 2*HQ is estimated as the whole body skeleton muscle/fat‐containing compartment. PDV is portal‐drained viscera. Stacked bars. Positive values within 95% CI indicate a net release of lactate, while negative values indicate a net uptake. Control *n* = 9 Sepsis *n* = 13. Data are expressed as mean with 95% CI.

**FIGURE 8:**
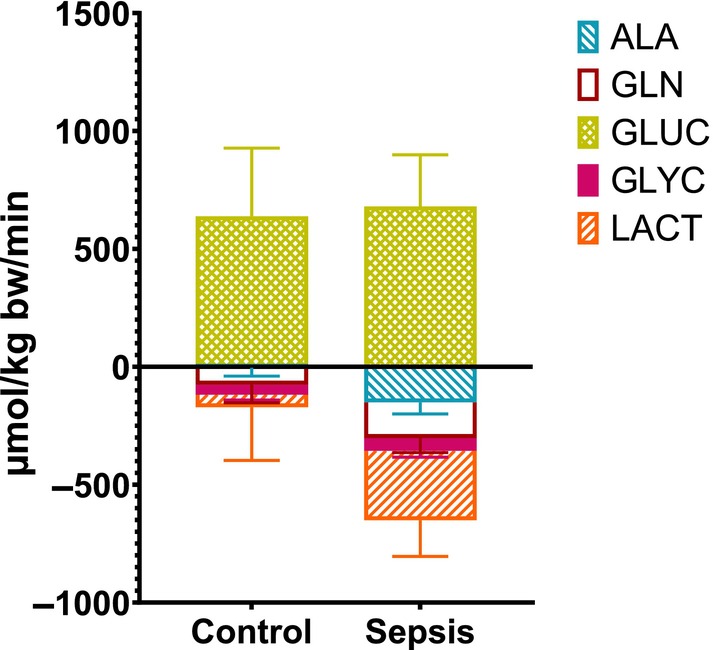
Liver glycogenic substrates uptake versus glucose release. Predicted net fluxes corrected for the carbon skeleton. GLUC is Glucose (6 carbon), GLN is glutamine (6 carbon), ALA is alanine (3 carbon), Glycerol (3 carbon), and LACT is Lactate (3 carbon). Stacked bars. Positive values within 95% CI indicate a net release of lactate, while negative values indicate a net uptake. Control *n* = 9 Sepsis *n* = 13. Data are expressed as mean with 95% CI.

Figure 7 shows a comparison between the net released glycogenic substrates in the skeleton muscle/fat‐containing compartment (2*HQ) and the uptake in the splanchnic area (PDV + LIVER). It indicates that except for lactate, the PDV took up less amount of substrates, and the splanchnic area took up more substrates than the muscle/fat compartment provided. The total net amount of the measured glycogenic substrates that is taken up by the liver is in the sepsis group larger (Figure 8). In the sepsis group the glycogenic substrate uptake (corrected for the carbon skeleton of each substrate) is in balance with the liver glucose release.

## 4 DISCUSSION

In the acute phase of sepsis, a lower arterial glucose concentration suggests an increased whole body glucose demand. We observed that the splanchnic area was compensating by an increased net release, not caused by an increased net liver production but by a reduced utilization of glucose in the PDV. We found that increased uptake of glycogenic substrates into the liver does not result in increased net glucose production but was in balance with net glucose release. The glycogenic substrates were made available by the skeleton muscle/fat compartment, but in these young female pigs mainly gluconeogenic amino acids and not glycerol. No difference between the groups in either glycerol release from the hindquarter, or uptake at the liver was observed, indicating that adipose tissue in our model is not being mobilized adequately for gluconeogenesis. Our study shows unexpected transorgan physiological responses to sepsis in the pig.

### 4.1 Glucose kinetics in sepsis

In healthy pigs, the expected response to an increased glucose demand would be to deplete glycogen stores and then to break down adipose tissue, releasing glycerol and free fatty acids. We previously found that glycogen is rapidly depleted in critical illness, thereby greatly reducing the body's ability to maintain normoglycemia (Hagve et al., 2020). Previous studies on net fluxes of glucose and amino acids across the liver in a catabolic state have shown an increased release of glucose (Bruins et al., 2003; Hagve et al., 2015; Meinz et al., 1998), although it is important to note that these studies used a prolonged infusion of endotoxin to induce sepsis, which may not reproduce key components of the immune response to a bacteriologically induced septic infection (Buras et al., 2005). In the control group, a lesser amount of glycogenic substrates were taken up in comparison to the glucose net release, indicating that in the control group glycogen is still available.

Dysregulation of glucose homeostasis in sepsis is a phenomenon that has been recognized for decades. Most publications discussing this phenomenon focus on the hyperglycemic aspect of this, which is caused by a variety of mechanisms such as inflammatory damage to pancreatic beta cells and increased peripheral insulin resistance (Hughes et al., 2009; Van den Berghe, 2004). Due to the research and clinical focus on hyperglycemia in sepsis, strict glucose control using insulin was formerly the standard of care. Recent work, however, has shown that the focus on hyperglycemia in sepsis has not led to improved patient outcomes (Bateman et al., 2016; Brunkhorst et al., 2008; Chen et al., 2018; Kalfon et al., 2014). In particular, several large randomized controlled studies showed that tight glycemic control using insulin does not result in improved mortality but rather increases the likelihood of inducing hypoglycemia in this patient population (Bouillon, 2006; Brunkhorst et al., 2008; Kalfon et al., 2014). Therefore, the prevalence and danger of hypoglycemia in sepsis have become more widely recognized (Mitsuyama et al., 2022; Wang et al., 2021).

In the acute phase of sepsis, which was examined in the present study, we observed a declining blood glucose concentration, without an adequate glucogenic response by the liver to maintain normoglycemia. We hypothesize that in the acute phase of sepsis, there are several faulty mechanisms in both the mobilization of glucogenic substrates, particularly glycerol, as well as the utilization of these substrates for the production of glucose. A common complication of the septic state is primary adrenal insufficiency. This complication leads to a relative deficiency of glucocorticoids in circulation, which could potentially lead to a hypoglycemic state if untreated. The mechanism behind this, however, is thought to be due to a chronic overstimulation of the adrenal glands by adrenocorticotropic hormone (ACTH) in response to stress. In the acute phase, however, cortisol is in abundance through a series of complex biochemical pathways (Annane et al., 2017). After several days to weeks of high adrenal stimulation though, the adrenal glands experience depletion resulting in a cortisol deficiency. As this process generally requires days to weeks of exposure to stress, it is therefore not likely to be the cause of the dysregulated glucose response seen in our experiments (Téblick et al., 2022).

**FIGURE 9:**
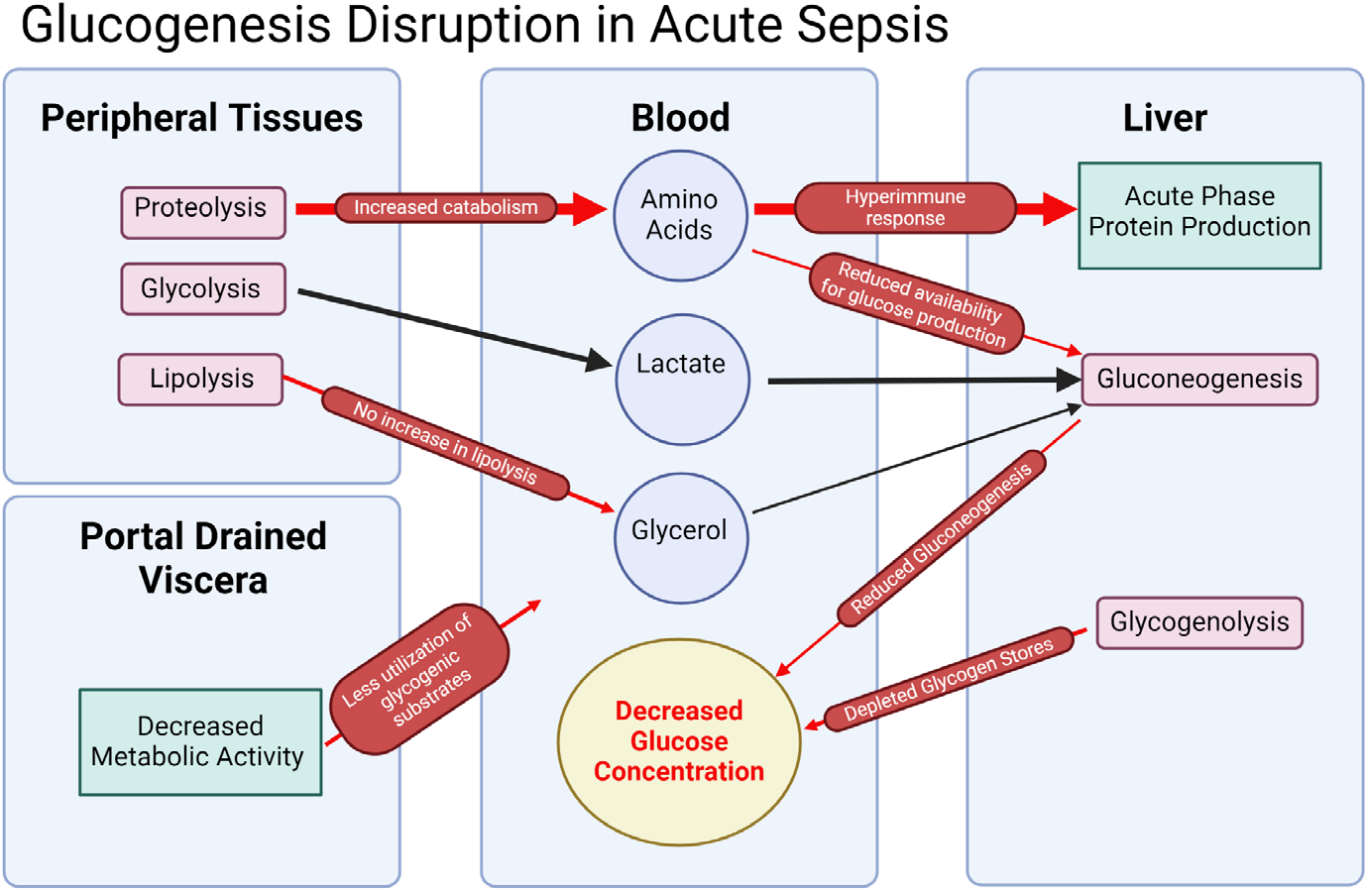
Proposed major glucogenic precursors and their disruption in sepsis.

As sepsis is a hypermetabolic state, glucose demand in many body tissues is greatly increased (Mészáros et al., 1988). Initially, the body is capable of responding to these increased glucose demands through both glycogenolysis and gluconeogenesis (Cherrington et al., 1984). Over time, however, hepatic glycogen stores are depleted, leading the liver to rely on gluconeogenesis to maintain normoglycemia. As will be discussed in subsequent sections, we hypothesize that several of the key metabolic pathways through which the body both synthesizes and utilizes glucogenic substrates are compensating for the high glucose demand in the acute phase of sepsis but failed to maintain normoglycemia. A graphic representation of the hypotheses of these physiological changes is depicted in Figure 9.

### 4.2 Lipid mobilization and utilization

In general, increased energy requirements during sepsis are met by increased lipolysis (Askanazi et al., 1980; Nordenström et al., 1982; Rittig et al., 2016), but this mainly is observed as a longer‐term characteristic of sepsis, and therefore may not accurately describe the conditions seen in early sepsis that were explored in the present model. The observed time effect on the glycerol systemic concentration, splanchnic uptake, and hindquarter release is likely related to the experimental conditions (Carpentier et al., 1980; Klein et al., 1991). Although we did not measure the glycerol whole‐body turnover, changes in glycerol plasma concentrations are highly correlated (Carpentier et al., 1980). The experimental conditions that mimic ICU conditions (extended postabsorptive state, fluid resuscitation) were also provided in the control group. Despite the septic state, these general ICU conditions are known to increase lipolysis in critically ill patients (Klein et al., 1991). Another factor that needs to be considered is that the total extent of lipolysis may be limited when the relative amount of body fat is low, like in pediatric burn patients (2%) (Wolfe et al., 1987). However, the young animals (age 2–3 month) in the present study should have already more than 20% of body fat (Filer Jr et al., 1960). Therefore, this is not likely to play a role. Also, the uptake by the liver did not differ between the groups. Despite the high variability, the results suggest no substantial extra demand by the liver for glycerol.

The hepatic metabolism of glycerol in sepsis has seen relatively little previous investigation. However, Leclercq et al. suggested a possible mechanism for the impairment of hepatic glucose production from glycerol (Leclercq et al., 1997). In the liver, glycerol is converted first into glycerol‐3 phosphate, and then into dihydroxyacetone phosphate by cytosolic glycerol‐3‐phosphate dehydrogenase as a precursor to glucose production. They showed a significant inhibition of cytosolic glycerol‐3‐phosphate dehydrogenase in the hepatocytes of mice treated with endotoxin, leading to a buildup of glycerol‐3‐phosphate in the liver. If this enzyme is inhibited in sepsis, glucose production from glycerol would likely be impaired.

### 4.3 Lactate kinetics

Elevated systemic lactate has been long established as being a principal component of sepsis. Broadly speaking, elevated lactate levels in sepsis are thought to be due to circulatory dysfunction resulting in inadequate oxygenation of tissue, resulting in anaerobic metabolism of glucose which releases large volumes of lactate as a byproduct (Hernandez et al., 2019). Due to the loss of efficiency that results from the anaerobic metabolism of glucose when compared to aerobic metabolism, relatively large quantities of glucose are required to maintain homeostasis in sepsis, which is likely a key driver of the glucose depletion observed in this study. This is reinforced by the significant increase we observed in serum lactate, which from our data appears to be primarily driven by increased release from the HQ. This increased lactate release from the HQ is most likely due to reduced peripheral tissue perfusion which is a common component of sepsis and correlated with disease severity (Gutierrez‐Zarate et al., 2023). In addition, several studies have established that in sepsis, blood glucose and blood lactate concentrations are inversely correlated, likely due to increased utilization for anaerobic metabolism (Kushimoto et al., 2020; Park et al., 2012).

As lactate is a byproduct of glucose metabolism in the tissue, it is also capable of being recycled by the liver back into glucose, and as one of the key glucogenic substrates, accounting for approximately half of glucogenic substrate in healthy fasted individuals (Katz & Tayek, 1998). In our study, we observed an increased production in lactate from the HQ, as well as increased uptake by the liver, although this liver uptake was inadequate to prevent the buildup of lactate in the blood. This inability to adequately clear lactate from the blood indicates that the metabolic pathways involved in the conversion of lactate to glucose are likely impaired in sepsis. While the details of lactate metabolism in sepsis are still not fully understood, several mechanisms by which the liver is unable to recycle lactate to glucose have been proposed. As with much of the dysfunction in sepsis, tissue hypoperfusion from circulatory dysfunction likely causes a portion of this hepatic impairment (Hernandez et al., 2019). It is important to note, however, that hypoperfusion is not required to produce impaired lactate clearance, with one study showing a 90% reduction in lactate clearance with the administration of lipopolysaccharide, which was not restored even with systemic resuscitation and adequate perfusion of hepatic tissue (Tapia et al., 2015).

### 4.4 Amino acid mobilization and utilization

In the acute phase of sepsis, it is well established that amino acid release from muscle breakdown is greatly increased (Bruins et al., 2003; Cho et al., 2020). In the present study, we explored the flux of the two most glucogenic amino acids, alanine and glutamine. These amino acids both showed an increased release from the hindquarter in the acute phase of sepsis, as well as an increased uptake in the liver, consistent with what has been established previously in the literature. Alanine is typically the amino acid released in the greatest quantities during muscle catabolism (Cahill Jr, 1976). In muscle, alanine comprises less than 10% of total amino acid content (Kominz et al., 1954), while making up approximately 30% of amino acid release from the muscle in the catabolic state in healthy subjects (London et al., 1965). The rest is supplied through the conversion of glucose to pyruvate, which is then transaminated to form alanine in the Cahill cycle (Felig, 1973). Glutamine is also more released by the skeletal muscle compartment in the sepsis group. However, the total amount is far less than the splanchnic area is taken up. Glutamine synthesis from glutamate and ammonia is considered mostly to be produced by the muscle. However, small amounts can be released by the lungs and brain (Newsholme et al., 2003). In healthy humans, glutamine production by the skeletal muscle compartment is considered to be comparable (in balance) with glutamate production in the liver (Deutz, 2008). Indeed also the glutamate HQ uptake and liver net release are lower in the septic state (Ten Have, Engelen, et al., 2017) (see supplementary data). Low systemic concentrations of glutamine in both groups are probably due to the general experimental condition's extended post‐absorptive state and resuscitation. However, it is commonly seen in ICU and trauma patients (Buchman, 2001).

Of particular importance to the present study, the uptake of alanine and glutamine by the liver was significantly increased. In most conditions, the liver is capable of converting these amino acids to glucose at a much higher rate than of the other amino acids, and alanine uptake contributes more to gluconeogenesis than all of the other amino acids combined (Felig, 1973). Despite the substantial increase in uptake of amino acids, particularly of alanine, there is still no significant increase in glucose production in the septic pigs in our study. This amino acid has several functions in the immune system, notably in the synthesis of acute phase proteins (Leonardi & Comolli, 1995) as there is a large increase in acute phase proteins during sepsis (Pierrakos & Vincent, 2010; Ron‐Harel et al., 2019), it is likely that alanine is also being utilized for the production of acute‐phase proteins, although further investigation in this area is warranted.

### 4.5 The role of the PDV on the availability of energy substrates

In the septic state, overall the PDV utilized less energy substrates compared to the control experimental state. This was expected considering the observed downregulation of protein metabolism in the gut, especially in the jejunum (Ten Have et al., 2019). Less energy was needed for the diminished protein metabolism. It is supported by the fact that changes in the citric acid cycle metabolites in the jejunum also were altered (Ilaiwy et al., 2019). Nevertheless, it results in higher energy substrate availability for the hyperactive liver, and due to the compromised liver gluconeogenesis, still net glucose release was observed by the splanchnic area and available for the extrahepatic tissues. Therefore, we hypothesize that the PDV plays a stabilizing, protective role in ensuring whole‐body glucose availability. It is unclear if energy substrates released from the microbiome like short‐chain fatty acids into the circulation also play a role in this phenomenon because they are also taken up by the liver in abundance (Kirschner et al., 2021; Mercer et al., 2020). Further investigation is needed into what their role could be.

### 4.6 Limitations

In this study, we explored the metabolism of several major gluconeogenic substrates in the acute phase of sepsis. A porcine model was used for this study due to the impracticality of sampling arterial and venous blood from the visceral organs in humans. Pigs may have a higher susceptibility to hypoglycemia in the septic state, as hyperglycemia is the more commonly observed glucose derangement seen in septic humans. However, in the ICU, glucose is infused in amounts that increases the prevalence for hyperglycemia in septic patients. Additionally, we explored only the acute phase of sepsis. It is likely that over longer timeframes, our subjects would have seen increased lipolysis and its contribution to gluconeogenesis, but this was outside of the scope of this study.

Also, the number of animals in this observational study is relatively small, considering the inherent variabilities in net flux measurements. However, with the chosen statistical approach, we could obtain a higher statistical sensitivity.

## 5 CONCLUSIONS

In the present study, we found that in the acute phase of sepsis, the liver does not increase its glucose production to maintain normoglycemia. This is the result of several metabolic failures, notably an inadequate mobilization of lipid stores and metabolic conversion of glycerol. The two main glucogenic amino acids, alanine, and glutamine, also do not provide an adequate substrate for gluconeogenesis. We observed a role of diminished PDV uptake of gluconeogenic amino acids that supports splanchnic glucose production. We hypothesize an important role of changed intestinal amino acid metabolism and breakdown of muscle proteins, but not of glycolysis to support splanchnic gluconeogenesis.

## AUTHOR CONTRIBUTIONS

The manuscript was drafted and prepared by RM and GTH. Data collection was made by GTH, ND, and ME. Data analysis was performed by RM, ND, and GTH. Manuscript edits and revisions were performed by SR, MH, ME, and ND.

## FUNDING INFORMATION

This study was funded by award number NIH R01GM084447 from the National Institute of General Medical Sciences and by award number S10RR027047 from the National Center For Resources.

## CONFLICT OF INTEREST STATEMENT

The authors have no conflicts of interest to disclose.

## ETHICS STAETMENT

Permission to conduct the study was granted by the Institutional Animal Care and Use Committee (IACUC) of University of Arkansas Medical Sciences (Little Rock, AR, USA). The research was conducted in accordance with the principles of AAALAC and in accordance with local statutory requirements.

## Supporting information


Figure S1.

## Data Availability

The data used in this manuscript are available upon reasonable request to the corresponding author.
