# Supplementary material for: The role of adipose and muscle tissue breakdown on interorgan energy substrate fluxes in a Pseudomonas aeruginosa induced sepsis model in female pigs
Source: Physiol Rep. 2025 Jan 3;13(1):e70129. doi: 10.14814/phy2.70129 (PMC11702427; doi:10.14814/phy2.70129)

Supplemental Figure 1. Actual and predicted net fluxes. Predicted net fluxes were calculated with predicted venous concentrations after ANCOVA analysis adjusted for the following independent variables: individual organ plasma flow, body weight, body temperature, and arterial concentrations of each animal.


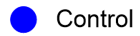

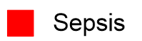


**Legend:**


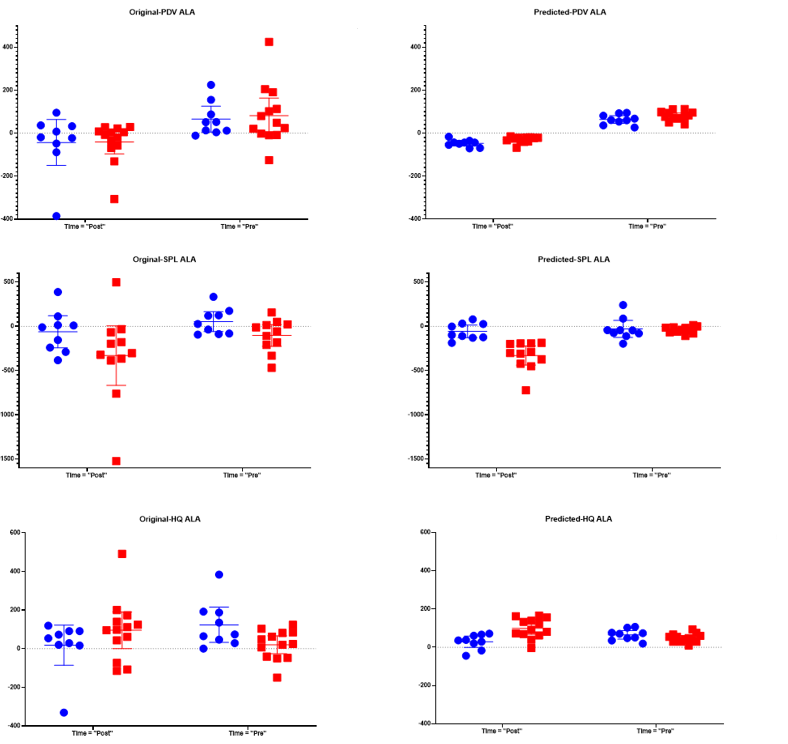


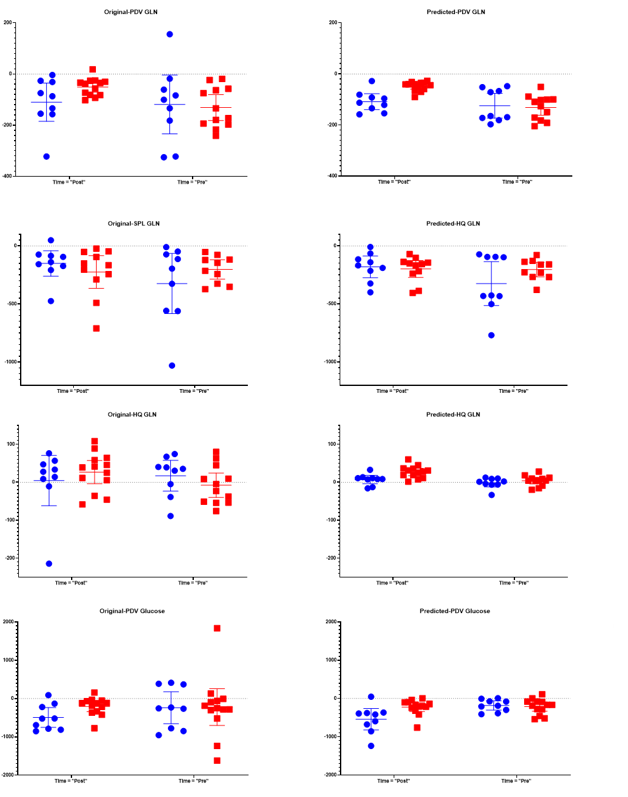


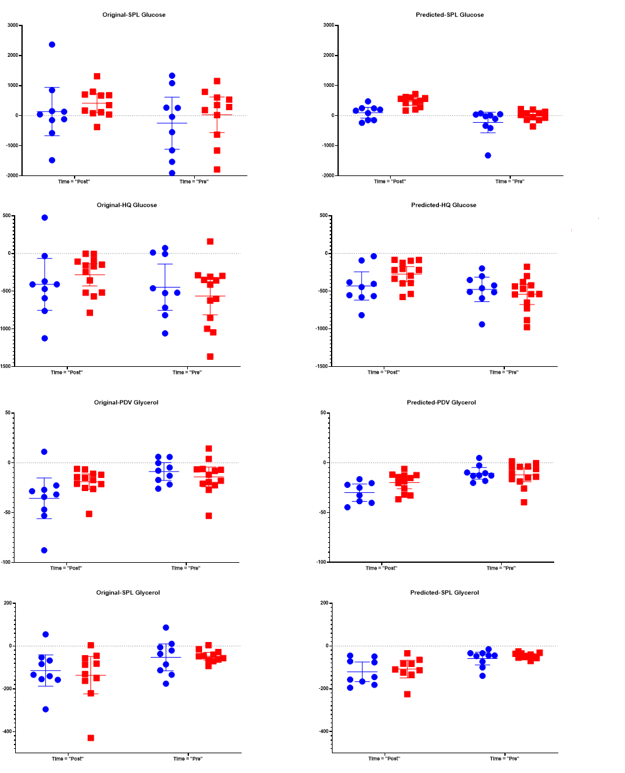


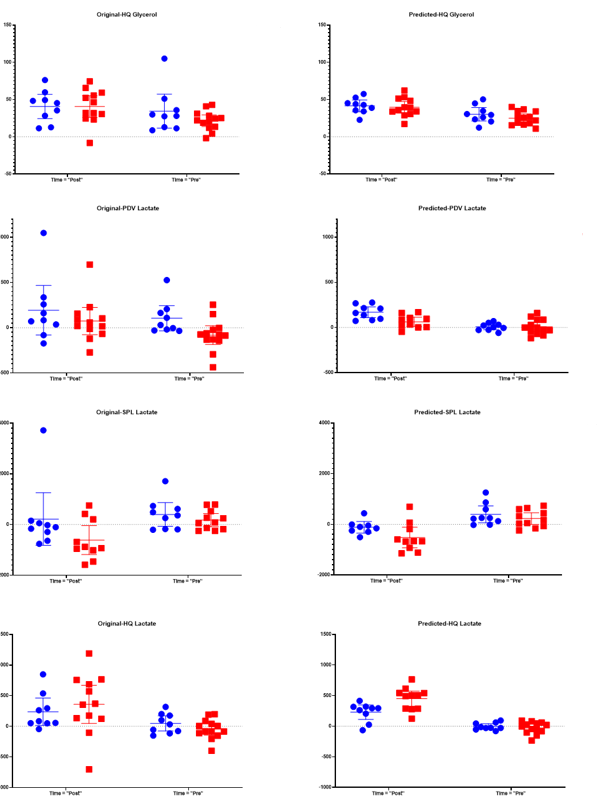

Supplement: Supplementary file 1 — Figure S1. [file PHY2-13-e70129-s001.docx]
